# Green Dialysis From the Indian Perspective: A Systematic Review

**DOI:** 10.7759/cureus.62876

**Published:** 2024-06-21

**Authors:** Surendra S Rathore, Kumari Nirja, Sunita Choudhary, Garima Jeswani

**Affiliations:** 1 Nephrology, Dr SN Medical College, Jodhpur, IND; 2 Physiology, Dr SN Medical College, Jodhpur, IND

**Keywords:** hemodialysis, global warming, green dialysis, eco-dialysis, dialyzer reuse, climate change

## Abstract

Global warming and climate change represent the most significant threats to humanity in the 21st century, both of which are manmade catastrophes. Addressing climate change requires corrective action across all aspects of modern human life and work, including the medical field. Among healthcare sectors, dialysis units stand out as major contributors to plastic waste and excessive water consumption. It is imperative for hemodialysis units to lead by example in the judicious use of natural resources.

This systemic review is aimed to establish a bare minimum of recommendations for environmental sustainability across Indian dialysis units, and to guide future initiatives to reduce the environmental impact of dialysis process. A literature search was conducted on PubMed, and Google to retrieve articles or studies related to green dialysis. The predefined keyword search yielded a total of 291 studies. A total of 54 studies and articles which were relevant to study question, and fulfilled inclusion criteria, were retrieved and analyzed to form opinions on the implementation of green dialysis initiatives from an Indian perspective.

Green dialysis initiatives are much-needed reforms to be adopted by the Indian dialysis community. Through careful planning and minimal efforts, substantial amounts of water used in hemodialysis can be conserved and repurposed for other hospital activities. Similarly, the vast majority of discarded plastic waste can be recycled or reused. Despite controversy, reconsidering the risk-benefit aspects of dialyzer reuse is essential, particularly in the context of resource-limited developing nations like India.

## Introduction and background

The environmental challenges of the 20th century have left an indelible mark on our planet, with mankind's relentless exploitation of natural resources resulting in devastating consequences. Now, as we confront the stark reality of climate change and global warming, we must reckon with the profound impact of our actions on the health of our planet and its inhabitants [1]. At the forefront of this battle are healthcare systems, whose facilities too often contribute to environmental pollution and waste generation. For instance, a report revealed that the National Health Services (NHS) in the United Kingdom accounted for a staggering 25% of all public sector carbon emissions, underscoring the significant environmental footprint of healthcare services [2]. Among these, dialysis programs stand out as one of the most significant contributors to medical waste generation [3-6]. While these programs have undoubtedly saved countless lives, they come at a steep environmental cost. Hemodialysis, in particular, is a resource-intensive intervention, consuming copious amounts of water and electricity while generating substantial plastic waste [5].

While efforts have been made globally to mitigate the environmental impact of hemodialysis [7], the Indian nephrology community has yet to fully recognize the magnitude of this issue. It is imperative that we engage in open dialogue, meticulous planning, and quantification of the dialysis-related water and waste problem to implement indigenous solutions that prioritize eco-friendliness. By proactively addressing these challenges, we can safeguard both the health of our planet and the well-being of future generations.

Due to the scarcity of literature on green dialysis initiatives from an Indian perspective, we are conducting a systematic review to address the following questions: What is currently known about green dialysis? What are the key components of eco-friendly dialysis initiatives, and what steps can be taken to make hemodialysis more environmentally friendly? By synthesizing the existing literature, we aim to offer insights and recommendations that are particularly relevant to the Indian context.

## Review

Methods

A literature search was conducted on PubMed, and Google using the following keyword combinations: Green dialysis; Eco dialysis; Water wastage and dialysis; Dialysis flow and dialysis. Inclusion criteria were only studies written in the English language and studies with full-text availability were included. Exclusion criteria were studies written in languages other than English and studies with unavailable full text were excluded.

Results

The predefined keyword search yielded a total of 291 studies and articles. Out of these, 188 studies were deemed irrelevant to the study question. Thirty-three studies were excluded for not being in the English language, and an additional 16 were excluded due to unavailability of full text for interpretation. Subsequently, the remaining 54 studies and articles were retrieved and analyzed to form opinions on the implementation of green dialysis initiatives from an Indian perspective.

Discussion

Environmental Impact of Dialysis

A single session of conventional hemodialysis produces approximately 2 kg of potentially contaminated waste per session [8], exacerbating the burden on our already strained ecosystems. Moreover, the per capita carbon footprint of dialysis patients is profound, surpassing even the average annual emissions of the general population in some regions [9-11]. For example, in Australia, a carbon footprint study of a suburban satellite dialysis service calculated the annual per-patient dialysis-generated carbon footprint of 10.2 tonnes CO2-equivalent (t CO2-eq), to be more than half the Australian mean annual per capita CO2 emission estimate of 18.8 t CO2-eq [11]. Given these data, it is plausible that dialysis services are likely to be the first target for the first wave of environmental belt-tightening measures to be taken by healthcare system regulatory agencies.

Effect of Climate Change on Kidney Health

Furthermore, our relationship with the environment is inherently reciprocal, with environmental changes influencing human health in profound ways. From the onset of renal disorders exacerbated by global warming and pollution to the emergence of chronic kidney diseases in regions exposed to high temperatures and agricultural toxins [12-15]. High temperatures, in conjugation with limited water intake, can cause multiple kidney hits which may lead to acute kidney injury (AKI), nephrolithiasis and urinary tract infections [12,16]. In recent years, epidemics of chronic kidney disease (CKD) have been identified in various hot regions of the world including the Indian subcontinent and Meso-American countries. Almost all cases occurred among outdoor farmers working for prolonged hours in fields and were not linked to diabetes, hypertension or glomerulonephritis [17,18]. Rather the occurrence of nephropathy was associated with exposure to high temperatures often in combination with agrochemicals, heavy metals, infectious agents, and genetic factors. While the pathogenesis is not fully clear, extended exposure to heat with subsequent chronic dehydration is considered as one pathogenetic mechanism [18-24]. The interplay between environmental factors and health outcomes is undeniable. As we strive to mitigate the environmental impact of healthcare practices, we must also recognize and address the broader environmental determinants of health, ensuring a sustainable and equitable future for all.

Magnitude of the Dialysis Population in India

Worldwide, about 10% of the population is affected by CKD [25,26]. While, in India the situation is even grimmer and the prevalence of CKD is estimated to be approximately 17% of the population [27,28]. The true burden of end-stage renal disease (ESRD) in India is not known, with few dedicated centres for care, lack of universal access to renal replacement therapy (RRT), and absence of a central registry. Even today, over 90% of patients requiring RRT in India die because of inability to afford care, and even in those who do start RRT, 60% stop for financial reasons. As per estimates, there are over 175,000 patients receiving dialysis in 2018 [29].

Addressing the Issue of Water Wastage in Dialysis Units

An essential component of any dialysis unit is reverse osmosis (RO) processed water, a vital element in the preparation of dialysate fluid by hemodialysis machines. This RO-processed water is derived from municipal drinking water, undergoing a meticulous purification process involving sand filtration, carbon filtration, deionization, reverse osmosis treatment, and ultraviolet light exposure to achieve its ultrapure state [30]. However, a significant challenge arises during this purification process: only 20-30% of the raw water can be converted into ultrapure water, with the remaining 70-80% being discarded as reject water (RW) [31,32]. While technically meeting the World Health Organization's standards for potable water, reject water is routinely disposed of due to its lack of chlorine, although it could serve various other purposes such as cleaning, laundry, etc.

The amount of water consumed in dialysis hinges on three key factors: (A) the method of discharge from reverse osmosis and the type of reverse osmosis system used, (B) the preparation of dialysate and reinfusate, and (C) the prescription of the dialysis session. According to estimates by Agar, a pioneer in green dialysis, a "standard" four-hour hemodialysis session consumes approximately 500 liters of water, a sharp contrast to the 2 to 3 liters per day required by the average individual [33]. Consequently, each dialysis session results in the wastage of 350-400 liters of potentially reusable water.

While data on this matter are limited and somewhat unreliable, a rough estimate of annual hemodialysis water consumption in India can be derived. Assuming that all 175,000 patients undergo a four-hour hemodialysis session twice weekly (the standard practice in India), the yearly water requirement for hemodialysis would be approximately 9 billion liters (175,000 x 102 x 500). Alarmingly, a significant portion of this water (6.3-7.2 billion liters) is needlessly channeled into sewage systems due to oversight and a lack of reuse initiatives. This estimate is likely conservative, as many patients require thrice-weekly dialysis, and the number of dialysis patients has surged in recent years due to the implementation of various free dialysis schemes by central and state governments [34]. This reality should serve as a wake-up call to the Indian nephrology community, particularly in regions, such as parts of western India, where water scarcity is a pressing concern. At our institution, water scarcity, especially during the summer months, is a recurrent challenge, often resulting in delays in dialysis shifts due to water unavailability. Urgent measures to mitigate water wastage in dialysis units are imperative.

Plastic Wastage

Hemodialysis treatment (and to a lesser extent peritoneal dialysis) produces an enormous quantity of waste [2,35]. Dialysis waste is divided into contaminated and non-contaminated waste. The non-contaminated waste (paper, jars, tubing and bags that were not in contact with biological fluids) can be disposed of as normal domestic waste is, while all materials that come into contact with body fluids are considered potentially contaminated and have to be disposed of separately [8,35]. While non-contaminated materials may be reused or recycled, it is generally believed that potentially contaminated material should not. It has been calculated that one hemodialysis session produces 5 to 8 kg of waste, approximately half of which is uncontaminated [3]. The weight of recyclable post-hemodialysis consumables varies between 2.5-2.9 kg/treatment, depending upon consumable choice [3,36]. Assuming a mean of 2.75 kg of recyclable waste, almost all of which currently ends up as infectious waste for landfill or incineration-total minimum annual Indian hemodialysis recyclable waste production alone is 4,90,87 tonnes (2.75×102×175000). A high percentage of hemodialysis plastic waste is composed of polyvinyl chloride (PVC); PVC is potentially recyclable, but mixing PVC with other plastic materials precludes recycling [2,8]. Furthermore, when PVC is discharged into a landfill or incinerated, it can release highly toxic dioxins and chlorinated organic compounds into the atmosphere and in the soil [37].

Regarding strategies for green dialysis, some highly recommended options for Indian Dialysis units are outlined below.

Water Conservation

Reuse of reject water: Reject water, the primary source of water wastage in dialysis units, represents a significant opportunity for conservation through reuse and recycling. In a single conventional hemodialysis session, an astonishing 350-400 liter of reject water is discarded. However, it's crucial to recognize that this reject water is not tainted or contaminated; rather, it is already highly purified tap water that has undergone a rigorous depuration cycle, effectively removing particulate matter, chlorine, chloramines, and other potentially harmful substances [33,38].

Despite its purity, this reject water is often deemed unsuitable for human consumption due to the absence of chlorine [33,39]. Yet, it has never encountered the dialyzer circuit or the patient and poses no greater infectious risk than tap water. Remarkably, its quality aligns with the standards set by the World Health Organization for potable water, with only a slight increase in conductivity [39]. Regrettably, due to prevailing ignorance, it is routinely wasted.

However, this reject water holds immense potential for alternative uses within hospital settings. It can be redirected to various departments, including the central sterilizing department for steam generation, hospital janitor stations and toilet flushers, hospital landscaping, community and aged care garden watering, sporting grounds, laundry facilities, and park maintenance [8,38].

Another effective strategy for conserving reject water is redirecting it to the main RO unit in a closed-loop circuit. Here, a meter within the RO system monitors the conductivity of recirculating water, automatically reinstating mains water flow if the conductivity of the recirculating reject water exceeds safe RO membrane extraction limits [33].

The benefits of reusing reject water are substantial. For instance, a dialysis service in the UK reported savings of up to 4 million liters of water per year through the implementation of an eco-friendly water system [40]. In the context of India, full recycling of reject water could save approximately 6.3-7.2 billion liters annually. Taking proactive measures, our institution has initiated steps to collect reject water from our dialysis unit, redirecting it for cleaning and laundry purposes.

Use of dry dialysis technology: While still in the experimental phase, the concept of wearable artificial kidneys and sorbent dialysis shows promise, particularly concerning water conservation. Typically, wearable artificial kidney dialysis consumes around 5-6 liters of water daily or 35-42 liters per week. Comparatively, when juxtaposed with the twice-weekly hemodialysis regimen of a patient, dry dialysis techniques could conserve up to 96% of water [2,31]. Additionally, sorbent dialysis systems boast advantages such as reduced power consumption, compact size, enhanced portability, and the capacity to produce purer water compared to conventional hemodialysis setups [8]. However, a notable challenge associated with sorbent-based dialysis technology is the disposal of used sorbents. Interestingly, these spent sorbents could serve as a potential nutrient-rich growth medium for salt-tolerant plant species like mangroves, seaweeds, or marine grasses [2].

Use of pre-prepared dialysate: In the United States, the NxStage machine (NxStage Medical Inc., Lawrence, MA, USA) is used for home hemodialysis patients [41]. Depending upon the dose of dialysis, the total fluid consumption with this system is between 25 and 80 liters of pre-prepared ultra-pure dialysate fluid only. In comparison to 500 liters of raw water consumed during a conventional hemodialysis session, this system can drastically reduce total water consumption. However, this system is still not available in India.

Reduce the dialysate flow rate: Reducing the dialysate flow rate (Qd) is a topic of interest in nephrology, as it has been conventionally set at 500 ml/min worldwide. However, there is limited research exploring the clinical and biochemical effects of lowering Qd below this standard threshold [39,42,43]. Several studies have investigated the impact of Qd changes on Kt/V, a marker of dialysis efficacy, with most finding no significant difference between lower (e.g., 400 mL/min) and standard (500 mL/min) Qd [44-47]. Notably, the influence of Qd on the clearance of small solutes such as urea and creatinine were observed in two studies, indicating dependency on Qd only when it falls below 200 mL/min [46,47]. Conversely, the clearance of beta-2 microglobulin was found to be unaffected by Qd [43,48]. Similarly, studies have not reported significant disparities in phosphorus removal between Qd rates of 400 and 500 mL/min [43,49].

In the late 90s, some centers experimented with higher Qd rates (700-800 ml/min) in attempts to enhance dialysis efficacy, particularly in patients with high blood flow, yielding modest improvements of less than 20% [45,50,51]. Notably, evidence suggests that lowering Qd may be feasible without notable negative clinical consequences in certain patient demographics, such as elderly individuals with low metabolic needs, poor vascular access, and suboptimal nutritional status [52]. Recent technological advancements have led to the development of new dialyzers with features like hollow fiber undulations, spacer yarns, and changes in fiber packing density, potentially improving flow distribution across the dialysate compartment. This innovation could theoretically reduce the necessity for high Qd rates. However, it's noteworthy that the effects of reducing dialysate flow rate below the conventional 500 mL/min have not been extensively studied with these newer dialyzers [39].

In conclusion, existing literature does not decisively demonstrate the impact of Qd changes on the clearance of uremic toxins or patient outcomes. Nonetheless, reducing Qd from 500 to 400 mL/min could significantly reduce water consumption during a standard four-hour hemodialysis session; hence, it might result in saving 72, 312 and 3744 liters of water a week, a month and a year, respectively. It would mean conserving 428 million liters of water annually, even on a nationwide scale like India [39]. However, further research across diverse patient populations is necessary to ascertain the safety and efficacy of Qd reduction in hemodialysis patients.

Effluent water management: Following treatment, effluent fluid is discharged into the sewage system directly from the ultrafiltration pipes of each machine. Termed as high-density wastewater (HD WW), this fluid has directly interacted with the patient's blood while traversing through hollow fiber dialyzers. Due to the potential risk of viral or bacterial contamination, it is systematically discarded. However, this fluid is rich in urea from dialysis patients and contains elevated levels of nitrites, phosphates, sulfates, ammonia, and total nitrogen, maintaining salinity at serum-equivalent levels.

It's worth noting that levels of organic matter and bacterial counts in HD WW remain within the parameters established by both the World Health Organization and the United Nations Food and Agriculture Organization (FAO) for agricultural usage [53]. In fact, HD WW is comparably less polluted than water currently utilized in agricultural practices [31]. Apart from a slight elevation in conductivity, all other components such as biochemical oxygen demand, nitrogen, chloride sulfate, and phosphorus concentrations remain within FAO standards. The bacterial count of the wastewater registers at 450 colony-forming units per milliliter, yet coliform organisms, notably Escherichia coli, are undetectable [53].

There is limited documentation on the reuse of HD WW. Tarras et al. documented a program in Morocco where HD WW was rendered suitable for agricultural application by subjecting the post-dialyzer effluent to a secondary reverse osmosis process [53]. However, an accompanying editorial raised concerns regarding the commercial feasibility of the additional power consumption necessary for regenerating the spent "effluent" dialysate [31].

Conservation of Consumable Waste

Plastic reuse and recycling: Currently, worldwide dialysis units produce approximately 750 million tonnes of plastic waste annually [54]. Similarly, our estimates suggest that Indian dialysis units generate around 9,78,000 tonnes of plastic waste each year. Traditionally, this waste is disposed of through landfilling or incineration [8]. However, a significant portion of these plastic consumables, such as Part A jars, plastic packaging, saline bottles, syringes, and caps, remain uncontaminated. This waste holds potential for reuse within the plastic industry through onsite reprocessing methods like sterilization, shredding, and packaging. Among these, the reuse of plastic jars is a widespread practice in developing countries, as it eliminates the need for additional sterilization units. Even the smallest items, like plastic bottle caps, hold ecological significance. For instance, onsite segregation and reuse of such items have demonstrated significant reductions in waste generation and cost savings in UK renal services [36]. Similarly, promoting the use of washable cotton bed sheets and gowns instead of single-use items can substantially reduce total plastic waste generation, a practice commonly accepted in developing countries like India. However, the environmental benefits of such practices must be balanced against the carbon footprint associated with washing, sterilization, and transportation.

Waste management approach: Effective waste management begins with the materials used in packaging, which often consist of relatively large amounts of plastic and paper, both easily recyclable. The key to successful reuse lies in appropriately sorting different items and preventing contamination of potentially recyclable materials. Achieving this requires proper training of dialysis staff in waste management procedures. Separation of waste should ideally occur at the point of generation [2,3,55,56]. It's essential to recognize that well-separated, non-contaminated "clean" material does not necessarily equate to recyclable material. Different plastics have limited compatibility for reusability, as demonstrated by experiences in Italy where less than 30% of materials separated from dialysis sessions were actually recyclable, with less than 10% undergoing the recycling process [35].

Dialyzer reuse: The re-use of dialyzers in plastic waste reprocessing programs for dialysis is a contentious issue, particularly regarding the re-use of dialyzers and blood lines. Repeated use of dialyzers significantly alleviates the burden of disposable waste in dialysis treatments by reducing the volume of infectious waste. Depending on the number of reuses, the amount of waste generated from single-use could be five to 30 times higher or more compared to that generated through re-use [39]. While dialyzer (and/or blood line) re-use is uncommon in most developed countries, it is nearly ubiquitous in Indian dialysis units [57,58]. Both proponents and opponents of dialyzer re-use present valid arguments.

Those against dialyzer re-use argue that it exposes staff to harmful chemicals such as formaldehyde and peracetic acid, increases the risk of hepatitis transmission due to improper labelling, and may lead to allergic reactions due to inadequate rinsing before subsequent use [59]. However, proponents argue that re-use significantly reduces the overall cost of dialysis procedures, which is a critical barrier in many third-world countries for providing universal renal replacement therapy [60]. Our extensive experience demonstrates that with careful staff training, the incidence of viral transmission is no higher than standard rates, and the likelihood of adverse reactions is minimal. Considering that dialyzers and blood lines are major contributors to dialysis-related plastic waste, it is time for the international nephrology community to reconsider the risk-benefit aspects of dialyzer reuse. Thus, re-use may still be acceptable from a broader environmental perspective, as it substantially reduces the volume of consumable waste.

The establishment of remote dialysis units: These are crucial, especially in countries like India where dialysis units are concentrated in urban areas while a significant portion of the population resides in rural areas. Dialysis patients often face arduous journeys for each session, with some travelling distances as long as 250 kilometers, particularly for patients with hepatitis B and HIV seropositivity, who may need separate machines not available in neighbouring districts. This logistical challenge leads to patients skipping dialysis sessions and experiencing health deterioration, exacerbating the carbon footprint due to extensive travel. To address this issue, it is imperative to establish small dialysis units at the township level, which can be electronically monitored by nephrologists working at medical colleges. This approach would enhance the quality of life for dialysis patients by improving adherence and simultaneously reduce the carbon footprint associated with the dialysis process.

Use of alternative energy sources: The adoption of alternative and renewable energy sources such as solar, wind, and wave power is gaining momentum worldwide. Solar energy holds particular promise for India, given its geographical location with most of the Indian subcontinent situated between the equator and the Tropic of Cancer. With abundant sunshine for the majority of the year, India presents an ideal environment for harnessing solar power [61].

One viable application lies in equipping the rooftops of hospitals and dialysis units with solar panels to generate electricity. This initiative is already gaining traction in India, primarily due to the escalating costs associated with conventional electricity. From personal observation, this endeavor shows significant potential for rapid expansion, driven by the compelling economics. The initial investment in establishing a solar energy system can be recouped within a few years, contingent upon the total energy consumption of the facility.

## Conclusions

The environment intricately intertwines with every aspect of our human existence. Safeguarding it is a collective duty we owe to future generations. The fate of our planet rests in our hands; even the smallest of steps can yield significant improvements to our ecosystem.

Among the myriad daily human activities, medical services play a pivotal role in shaping the current waste landscape. Dialysis therapies, in particular, stand out as one of the most resource-intensive medical technologies. Each session can generate up to 2 kg of potentially contaminated waste, alongside an equivalent amount of potentially recyclable materials, compounded by technological waste from dialysis machines. Furthermore, this process incurs substantial water and electricity consumption.

This systematic review endeavors to inspire the Indian nephrology community to contemplate the strides they can undertake to foster environmental well-being. To foster eco-friendliness within dialysis therapy, we propose a series of simple and cost-effective measures: First, newer, more efficient RO plants should be installed to curtail water consumption. Furthermore, for reject water management, systems for storing and reusing reject water should be introduced. Likewise, re-use of "household" hospital items such as linens and overcoats must be encouraged. A well-organized triage system for separating contaminated and non-contaminated materials will facilitate non-contaminated material recycling, such as plastics, papers, glass bottles, dialysate jars, and cardboard materials. We advocate enhanced utilization of solar energy so that dependency on fossil fuel may be minimized. Establishment of remote small-scale dialysis units is desirable to minimize resource consumption and logistical challenges.
